# The *Arabidopsis CURVY1* (*CVY1*) gene encoding a novel receptor-like protein kinase regulates cell morphogenesis, flowering time and seed production

**DOI:** 10.1186/s12870-014-0221-7

**Published:** 2014-08-27

**Authors:** Emma W Gachomo, Lyla Jno Baptiste, Timnit Kefela, William M Saidel, Simeon O Kotchoni

**Affiliations:** Department of Biology, Rutgers University, 315 Penn St, Camden, NJ 08102 USA; Center for Computational and Integrative Biology, 315 Penn St, Camden, NJ 08102 USA

**Keywords:** *CURVY1*, Cell morphogenesis, *Arabidopsis thaliana*, Distorted trichome, T-DNA knockout mutant, Actin bundle, Protein kinase, Seed production

## Abstract

**Background:**

A molecular-level understanding of the loss of *CURVY1* (*CVY1*) gene expression (which encodes a member of the receptor-like protein kinase family) was investigated to gain insights into the mechanisms controlling cell morphogenesis and development in *Arabidopsis thaliana*.

**Results:**

Using a reverse genetic and cell biology approaches, we demonstrate that *CVY1* is a new *DISTORTED* gene with similar phenotypic characterization to previously characterized *ARP2/3* distorted mutants. Compared to the wild type, *cvy1* mutant displayed a strong distorted trichome and altered pavement cell phenotypes. In addition, *cvy1* null-mutant flowers earlier, grows faster and produces more siliques than WT and the *arp2/3* mutants. The *CVY1* gene is ubiquitously expressed in all tissues and seems to negatively regulate growth and yield in higher plants.

**Conclusions:**

Our results suggest that *CURVY1* gene participates in several biochemical pathways in *Arabidopsis thaliana* including (i) cell morphogenesis regulation through actin cytoskeleton functional networks, (ii) the transition of vegetative to the reproductive stage and (iii) the production of seeds.

**Electronic supplementary material:**

The online version of this article (doi:10.1186/s12870-014-0221-7) contains supplementary material, which is available to authorized users.

## Background

In plants, cell shape patterning and growth are regulated by multiple genes that are mediated by actin and microtubule cytoskeleton-dependent trafficking pathways [1-3]. The combined activities of the cytoskeleton, endomembrane, and cell wall biosynthetic systems organize the cytoplasm and define the architectural cell patterning [1-3]. Genetic screens have identified a class of mutants known as *DISTORTED* mutants because of their significant actin-related cytoskeletal growth-associated phenotypic defects and overall distorted cell shape patterning and abnormal polarized growth (trichome, epidermis, cell-cell communication) [2,4-6].

Genetic analysis reveals that gene that function in signal transduction cascades controlling local actin polymerization through the ARP2/3 complex [7-10] and the SCAR/WAVE complex [5,11-18] regulate cell patterning/morphogenesis in plants. Most of this knowledge comes from studies of differently distorted trichome mutants generally characterized by irregular cell expansion and polarized growth [2,4,19,20].

In order to decipher the genetic basis of plant cell shape patterning and growth, we employed, in this study, a reverse genetic approach by screening the loss of gene expressions in Arabidopsis T-DNA knockout mutants to gain insights into the mechanisms controlling cell morphogenesis in plants. *DISTORTED* mutants are known to display a dramatic cell shape alteration in comparison to wild type plants. The overall cell (trichome, pavement cell, root system) morphology of *DISTORTED* mutants has been well studied [21]. The *DISTORTED* genes have been reported to function in signal transduction cascades that control actin cytoskeleton assembly through WAVE/SCAR2-ARP2/3 pathway [2,3,20,21].

In this manuscript, we describe a new *DISTORTED* gene termed *CURVY1* (*CVY1*) that encodes a member of the receptor-like kinase (RLK) superfamily. Protein kinases are generally involved in perception of general elicitors initiating signal transduction cascades regulated by protein phosphorylation [22] to activate downstream responses that include the production of reactive oxygen species, ethylene biosynthesis, activation of a MAPK cascade, activation of abiotic or defense gene expression and other biological processes [23-26]. In addition, RLKs have also been recently related to the regulation of unidimensional cell growth, response to nitrate, and transferase activities in eukaryotes [22]. several protein kinases and their biological phosphorylation processes are still largely uncharacterized in *Arabidopsis thaliana*. Among the protein kinase genes, the *CURVY1* (*CVY1*) gene appears to have a unique function related to cell morphogenesis, as *cvy1* mutant displays phenotypes similar to distorted *SCAR/WAVE* and *ARP2/3* mutant cell morphologies [2,4,16,27]. Using a reverse genetic approach, we examined and characterized a SALK_T-DNA knockout *curvy1* mutant (*cvy1*) with respect to cell morphogenesis and growth phenotypes. Knockout mutation in *CVY1* caused severe trichome growth defects with relatively mild effects on overall shoot development, demonstrating that *CVY1* functions in polarized cell growth and cell shape patterning. In addition, the work demonstrates that *CURVY1* represents a novel receptor-like kinase that regulates trichome, pavement cell morphogenesis and cell wall biogenesis among other interesting phenotypic features and might function in signal transduction cascades that control local actin assembling through the SCAR2/WAVE-ARP2/3 pathway.

## Results and discussion

### Genetic and phenotypic characterization of *curvy1* mutant

**Figure 1:**
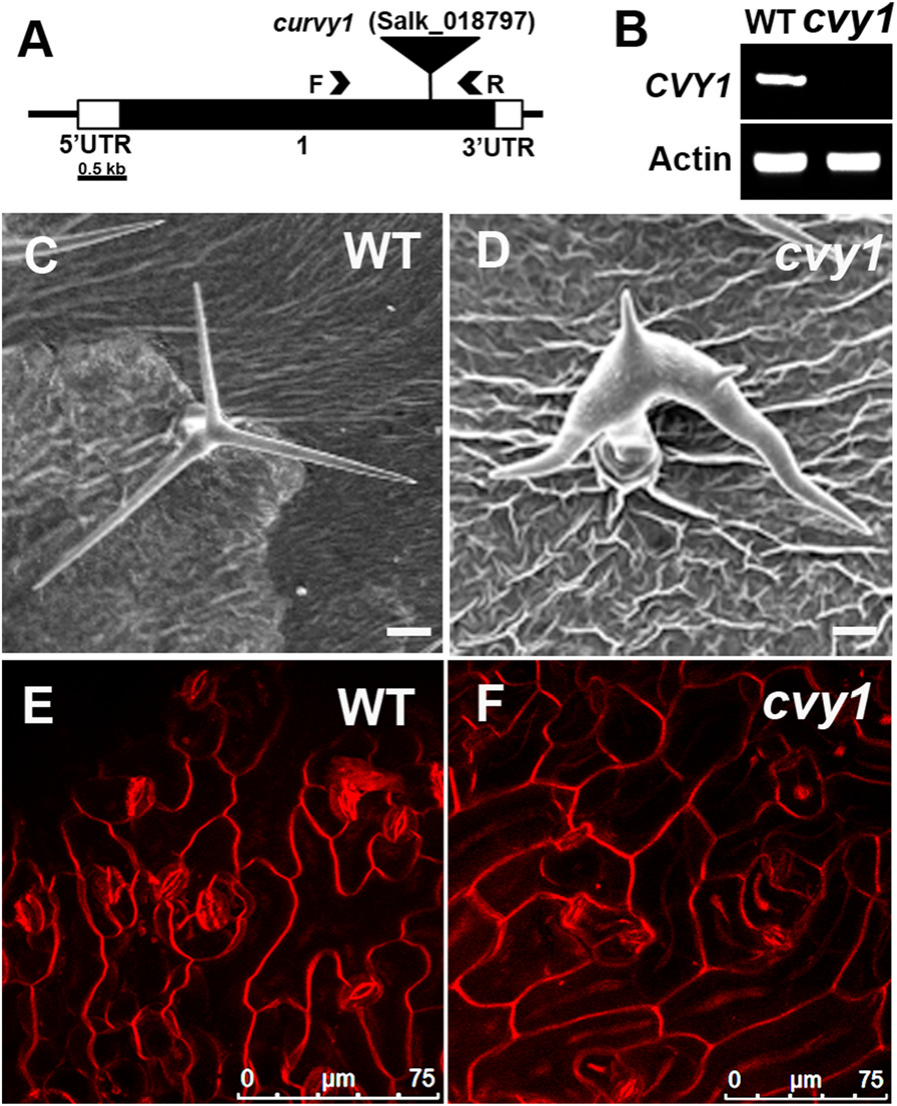
**Physical map of** ***CVY1*** **gene knockout and phenotypic characterization of** ***cvy1*** **mutant. (A)** The *CVY1* gene with the positions of the exon (numbered black rectangle) of the gene represented. The 5’ and 3’ untranslated regions are depicted in white rectangles. The location of the Salk T-DNA insertion is shown using an inverted black triangle. The names and locations of primers used for RT-PCR analysis are also indicated. Bar = 0. 5 kb. **(B)** The T-DNA insertion causes a knockout expression of the gene. The quality of the RNA and the loading control was assayed by monitoring ACTIN gene expression. **(C and D)** SEM images of upper developing leaves, showing a mature trichome with three branches in wild type **(C)** and strong distorted trichome in *cvy1* **(D)** plants. **(E and F)** Confocal images of pavement cell shape pattern of 12 days old WT **(E)** and *cvy1* **(F)** using lipophilic dye, FM464. Bars = 50 μm **(C, D)**.

To investigate the role of *CURVY1* in regulating cell morphogenesis in plants, we initiated a reverse genetic analysis of the gene using the Salk collection of Arabidopsis T-DNA knockout lines of our in-house Arabidopsis seed stock library. *CURVY1* is here shown to be important not only for polarized cell growth and trichome morphology but also other biological processes including flowering time and seed production. Our data reveals that mutations in *CURVY1* gene results in strong-distorted trichomes that are similar to the *SCAR/WAVE* and *ARP2/3* mutant phenotypes [2,5,7-18]. To our knowledge, this is the first time that *CURVY1* has been shown to control cell morphology/patterning (Figure 1). In addition, we investigated the role of *CURVY1* in other biological processes. We employed a reverse genetic approach using the Arabidopsis T-DNA SALK lines mediating loss of function of *CURVY1* gene to examine *curvy1*-knockout phenotypes. The SALK_018797 (*curvy1*) line harboring a T-DNA insertion in the only exon of *CURVY1* gene map (Figure 1A) was selected and confirmed as null mutant with loss of *CVY1* function. We confirmed the location of the T-DNA using the T-DNA-specific oligonucleotide primer LB1 and the *CVY1*-specific primer (Table 1) and examined the *CVY1* mRNA transcript levels in wild type and *cvy1* mutant using RT-PCR. As shown in (Figure 1B), the T-DNA insertion caused a knockout of the *CVY1* gene in *cvy1* mutant background. The mutation caused significant distortion of trichomes (Figure 1C, D, Table 2) and altered pavement cell morphology (Figure 1E, F, Table 3) compared to wild type. The *cvy1* cell patterning (trichomes, epidermal cells) is not obviously different from previously characterized *arp2/3* (*arpc2*, *arpc4*) distorted mutants (Tables 2 and 3). The tissue specific expression pattern of *CVY1* (Additional file 1: Figure S1) is consistent with Genevestigator microarray data [28]. The *CURVY1* gene is ubiquitously expressed in all tested tissues, but particularly high in polarized cells/tissues such as the trichome, root, root tip, and hypocotyls (Additional file 1: Figure S1), suggesting its importance in plant cell morphogenesis and polarized cell growth.

**Table 1:** **Sequences of oligonucleotide primers used in this study**

**Name**	**Primer sequence**	**Description**
*CVY1*-F1	5’TGCGATGGAGACTGTTTCTCGTGT3’	For RT-PCR
*CVY1*-R1	5’ATCAGAGTTTAACCTCGTGGCGGT3’	For RT-PCR
TDNA-LB	5’CCGTCTCACTGGTGAAAAGAA3’	For TDNA insertion
*CRV1*-F2	5’ATCAT*CCCGGG*TATCTTCTCCGAATATAGACT3’	For complementation test (SmaI site italicized)
*CVY1*-R2	5’CAATTG*CCCGGG*ATATATAATTTAAGCTTCTTTGT3’	For complementation test (SmaI site italicized)
*Act2*-F	5’GCGGATCCATGGCTGAGGCTGATGATATTCAACC3’	For RT-PCR
*Act2*-R	5’CGTCTAGACCATGGAACATTTTCTGTGAACGATTCC3’	For RT-PCR

**Table 2:** **Comparative quantitative phenotypic analysis of** ***cvy1*** **trichomes to well characterized** ***arp2/3*** **trichome mutants**

**Trichome**	**WT**	***curvy1***	***arpc2 (dis2)***	***arpc4***
Branch 1 (μm)	286 ± 31 (n = 16)a	82 ± 27 (n = 10)d	87 ± 31 (n = 14)d	78 ± 26 (n = 12)d
Branch 2 (μm)	256 ± 50 (n = 16)b	30 ± 10 (n = 10)e	29 ± 8 (n = 14)e	28 ± 12 (n = 12)e
Branch 3 (μm)	196 ± 46 (n = 16)c	22 ± 12 (n = 10)f	18 ± 7 (n = 14)f	20 ± 8 (n = 12)f

The numbers in the parentheses indicate the number of samples analyzed. Mean values with different letters are significantly different from each other, and mean values with the same letter in the group are not significantly different (P <0.05).

**Table 3:** **Comparative quantitative analysis of** ***cvy1*** **pavement cell shape phenotype to well characterized** ***arp2/3*** **pavement cells**

**Pavement cell**	**WT**	***curvy1***	***arpc2 (dis2)***	***arpc4***
Size (μm^2^)	2.10 ± 0.6 (n = 25)a	1.56 ± 0.3 (n = 24)d	1.70 ± 0.61 (n = 20)d	1.62 ± 0.31 (n = 28)d
Circularity*	0.25 ± 0.06 (n = 25)a	0.38 ± 0.05 (n = 24)d	0.34 ± 0.06 (n = 20)d	0.30 ± 0.03 (n = 28)d

The numbers in the parentheses indicate the number of samples analyzed. Mean values with different letters are significantly different from each other, and mean values with the same letter in the group are not significantly different (P <0.05). *Circularity describes the cell shape complexity.

### CURVY1 controls cell morphogenesis in plants

**Figure 2:**
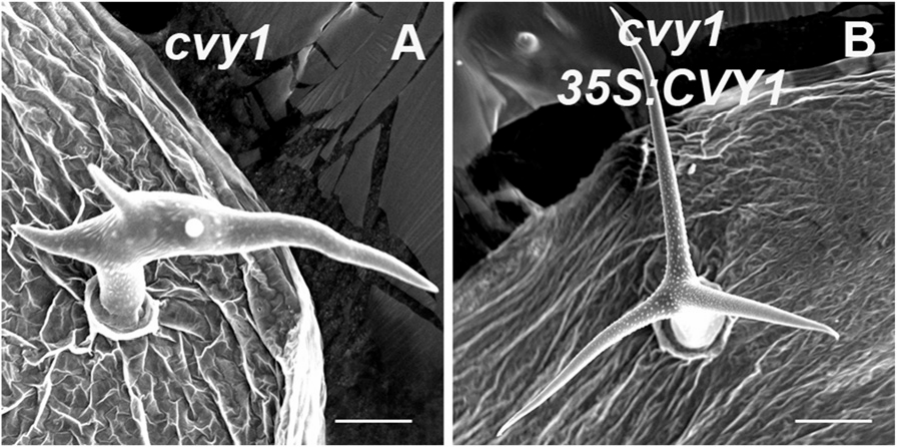
**Overexpression of** ***CVY1*** **gene rescues the** ***cvy1*** **trichome phenotype in a complementation test. A)** Distorted trichome phenotype of *cvy1* mutant. **B)** The distorted trichome phenotype in *cvy1* mutant is perfectly rescued by *35S:CVY1* gene expression.

We confirmed that *cvy1* morphological phenotype was indeed caused by the described T-DNA insertion by constitutively overexpressing *CVY1* gene in *cvy1* mutant background. This complementation functionality test was performed by using *Agrobacterium tumefaciens* mediated transformation to introduce the 35-promoter-*CVY1* transgene into *cvy1* plants [29]. As expected, overexpression of *CVY1* in *cvy1* mutant background was sufficient to rescue the *cvy1* phenotype (Figure 2A, B), demonstrating that *CVY1* gene knockout is indeed responsible for the phenotypic characterization in *cvy1* mutant phenotype, and thus providing further confirmation of the correct genetic characterization of *CURVY1* as a new “*DISTORTED*” gene. The T-DNA (SALK_018797) causing knockout mutation in *CVY1* (At2g39360) is also present in *MIR156A* (At2g25095) gene that targets SPL3. However, we ruled out the possibility of *cvy1* mutant phenotypes being caused by a plausible insertion on *MIR156A* gene because homozygous *mir156A* mutant does not have distorted trichome phenotype and the overall phenotypic complementation tests (trichome phenotype, flowering time, seed production and hypocotyl gravitropism) excluded the implication of *MIR156A* mutation in the observed/described *curvy1* phenotypes (Table 4).

**Table 4:** **Overexpression of** ***CVY1*** **gene rescues the overall** ***cvy1*** **phenotypes in complementation tests**

**Phenotypes**	**WT**	***curvy1***	***cvy1_35S:CVY1***
Flowering time (in number of rosette leaves)	14.0 ± 1.5 (n = 22)a	10.0 ± 1.1 (n = 28)b	15.5 ± 2.0 (n = 12)a
Number of siliques/seed production at 31 days	12.5 ± 2.0 (n = 22)a	45.0 ± 5.0 (n = 28)b	14.0 ± 4.0 (n = 12)a
Dark grown phenotype	GG (n = 22)	LGG (n = 28)	GG (n = 12)

Flowering time, siliques/seed production and dark phenotypes of *cvy1* mutant were rescued by *35S:CVY1* gene expression in a complementation test. Numbers in the parentheses indicate the number of samples analyzed. Mean values with different letters are significantly different from each other, and mean values with the same letter in the group are not significantly different (P <0.05). GG = Grow against gravity; LGG = Loss of growth against gravity.

**Figure 3:**
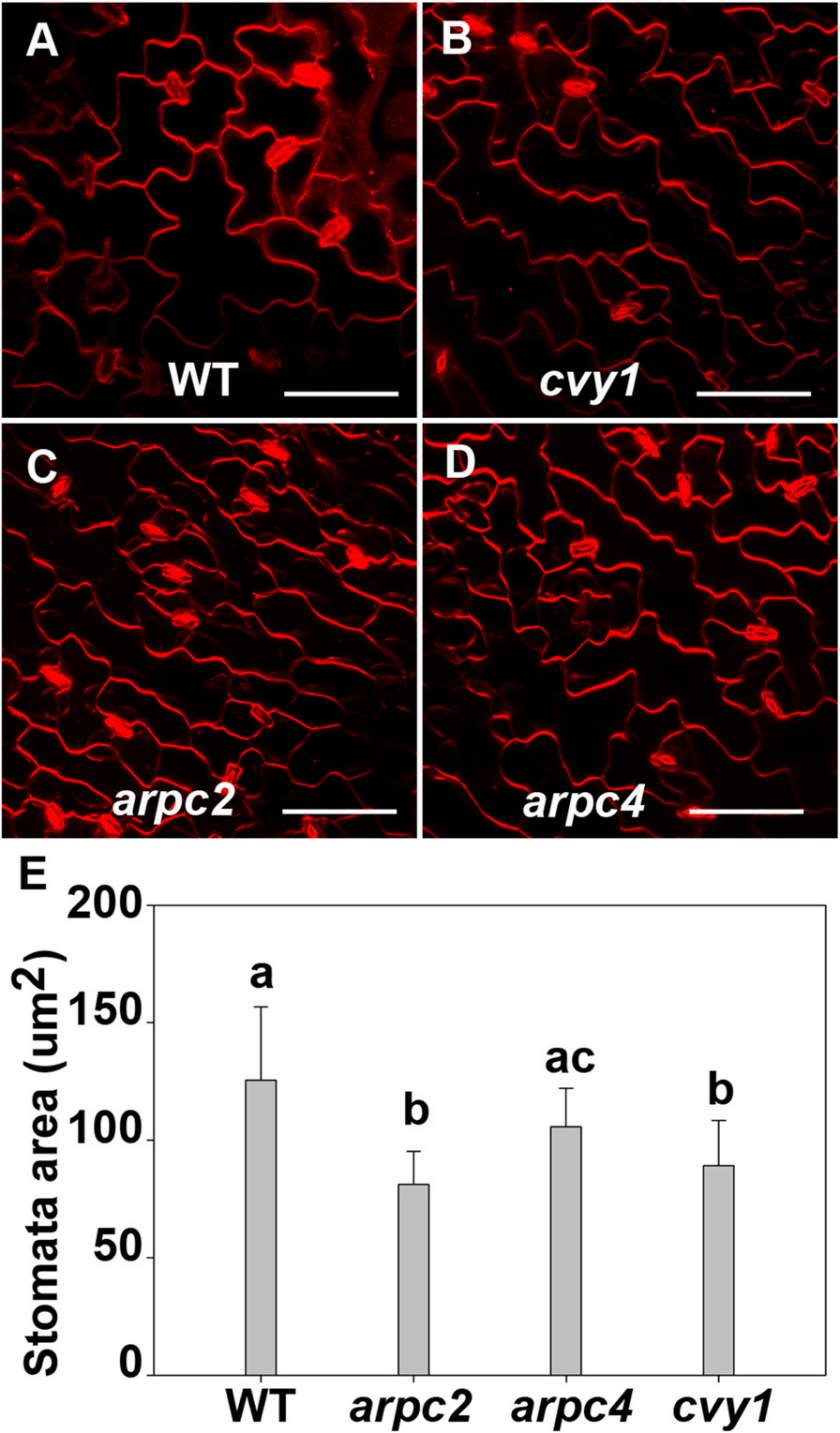
***curvy1*** **cell shape phenotype is indistinguishable from** ***arp2/3*** **cell shape mutants. (A-D)** Wide-field fluorescence images of fields of cotyledon epidermal pavement cells of wild-type **(A)**, cvy1 **(B)**, arpc2 **(C)** and arpc4 **(D)**. **(E)** Wild type-stomata are bigger than the mutant-stomata. Stomata mean values with different letters are significantly different from each other, and mean values with the same letter in the group are not significantly different (P <0.05). Bars = 50 μm.

Trichome branch length assay and pavement cell phenotypes are generally the most sensitive assays to describe phenotypic similarity among different distorted mutants [2,20]. The trichome phenotypes (Table 2) of *curvy1* mutants were indistinguishable from the well characterized *ARP2/3* distorted mutants (*ARPC2* and *ARPC4*). In addition, *cvy1* shape complexity of pavement-cells was significantly reduced compared to WT (Figure 1E, F), but was also indistinguishable from *arpc2* and *arpc4* pavement cells (Figure 3A-D, Table 3), suggesting a conserved cell shape regulatory relationship between *CURVY1* and *ARP2/3* in plants. The data suggests that *CURVY1* belongs to the “distorted group” of genes. *ARP2/3* gene mutations are associated with actin cytoskeleton defects [2], suggesting that *CURVY1* might regulate cell morphogenesis through signal transduction cascades that control local actin assembly through the ARP2/3 complex or the SCAR/WAVE complex [2,20]. In addition, we scored the stomata surface areas and found WT-stomata overall to be bigger than the stomata of mutants (Figure 3E). We analyzed the growth of wild type, *curvy1* mutants and the well characterized *arp2/3* (*arpc2*, *arpc4*) mutants under latrunculin B (LatB), an actin filament depolymerization drug [30]. The wild type (n = 22 seedlings), *curvy1* (n = 28 seedlings) and *arp2/3* (n = 12 seedlings) were affected by LatB (5 nM) treatment. However, we observed a significantly stronger effect of LatB on *curvy1* mutants that was indistinguishable from the effect of LatB on *arp2/3* (*arpc2* and *arpc4*) mutants (Table 5), supporting the notion that *CURVY1* might regulate cell morphogenesis through actin cytoskeleton networks [2,30]. To further make the link between CURVY1 and the actin cytoskeleton, we tested the sensitivity of *cvy1* rescue lines to the actin depolymerization drug LatB. As expected, the effect of LatB on *cvy1* rescue lines (n =20 seedlings) were similar to that of the wild type (n = 22 seedlings). Overall, these data demonstrate that CURVY1 regulates cell morphogenesis through actin cytoskeleton functional network.

**Table 5:** **The effect of latrunculin B (LatB) on wild type** ***arp2/3*** **and** ***cvy1*** **seedlings**

**Treatment**	**Root length (mm)**			
	**WT**	***curvy1***	***arpc2 (dis2)***	***arpc4***
Control	15.5 ± 0.5 (n = 22)a	15.0 ± 0.3 (n = 28)a	10.0 ± 0.6 (n = 12)b	9.0 ± 0.3 (n = 12)b
LatB (5 nM)	7.0 ± 0.05 (n = 22)c	5.0 ± 0.05 (n = 28)d	4.5 ± 0.06 (n = 12)d	4.0 ± 0.03 (n = 12)d

The numbers in the parentheses indicate the number of samples analyzed. Mean values with different letters are significantly different from each other, and mean values with the same letter in the group are not significantly different (P <0.05). The data was generated from vertical plate grown seedlings for 12 days under continuous illumination.

### *CURVY1* encodes a member of the receptor-like kinase (RLK) protein family

The RLKs are integral plasma membrane associated proteins with an extracellular domain that mainly binds to a carbohydrate, a transmembrane domain, and an intracellular Ser/Thr kinase domain [31]. Overall, plant RLKs have been reported to regulate various signaling pathways, including meristem function, brassinosteroid perception, floral abscission, ovule development and embryogenesis, plant defense, and plant morphology [32]. Previous studies showed that selected members of Arabidopsis *CrRLK* gene family including FERONIA (FER: At3g51550) [33-36], THESEUS1 (THE1: At5g54380) [37], HERCULES1 [35], ANXUR1 and ANXUR2 (ANX1 and ANX2) [38,39] regulate cell growth processes in different tissues under different development conditions. Likewise, CURVY1 has been found to control plant cell morphology and overall growth including flowering time, cell polarity, and actin cytoskeleton network.

**Figure 4:**
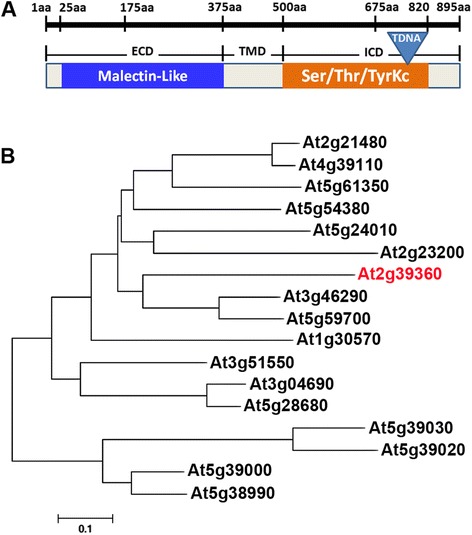
**CURVY1, a member of Arabidopsis CrRLK1-like family. (A)** The CURVY1 protein with all structural features of CrRLKL1 protein family is depicted. The position of T-DNA is depicted on the map. ECD, extracellular domain; TM, transmembrane domain; ECD, intracellular domain; Ser/Thr/TyrKc, serine/threonine/tyrosine kinase catalytic domain. **(B)** A phylogeny tree based on the full-length amino acid sequence of the Arabidopsis members of CrRLK1-like family. CURVY1 (in red) belongs to the largest subclade composed of well characterized RLK members such as HERCULES1 (HERK1: At3g46290), HERCULES2 (HERK2: At1g30570) and THESEUS1 (THE1: At5g54380).

*CURVY1* gene encodes a receptor-like kinase (RLK) that belongs to the *Catharanthus roseus* RLK (CrRLK)-like family [40,41]. RLKs represent a large diverse family of proteins with approximately 600 members in *Arabidopsis thaliana* [42]. However, the CrRLK-like family comprises a conserved extracellular carbohydrate-binding malectin-like domain [40] with 17 members in Arabidopsis (Figure 4) and 20 in rice [40]. As expected, CURVY1 displayed all protein features (malectin-like domain, serine/threonine-protein kinase active site, protein kinase catalytic domain) of well characterized CrRLK-like family (Figure 4A, [41]). Interestingly, all 17 Arabidopsis members of *CrRLK* gene family are structurally well conserved. They are exclusively made of a single exon flanked with a variable UTR length structure at both 3’ and 5’ ends. Nine out of the 17 Arabidopsis members of CrRLK1-like gene family are located on chromosome 5, three on chromosome 2 and 3 respectively and one on chromosome 1 and 4 respectively (Figure 4B). Phylogenetic analysis revealed four subclasses with CURVY1 belonging to the larger subclass composed of 10 members including the well characterized THESEUS1 (THE1: At5g54380) and HERCULES1 (HERK1: At3g46290) (Figure 4B). These four subclasses suggest a diversification of Arabidopsis CrRLK1-like proteins based on functional specifications (Figure 4B).

### Actin bundles are disorganized in *curvy1* epidermal cells

**Figure 5:**
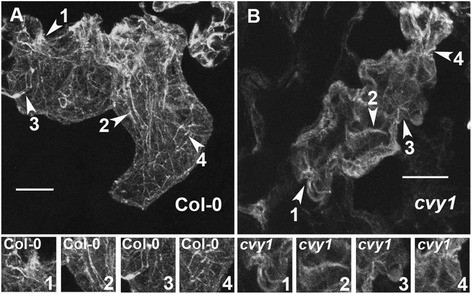
**Knockout** ***cvy1*** **null mutant displays reduced and disorganized actin bundles. (A and B)** Actin organization in wild-type and curvy1 pavement cells was visualized using fluorescent phalloidin as previously described [2]. Depicted regions (arrow heads with numbers) of WT and *curvy1* pavement cells were magnified in the bottom panels to display the actin bundles in respective genotype backgrounds. Bars = 10 μm.

**Figure 6:**
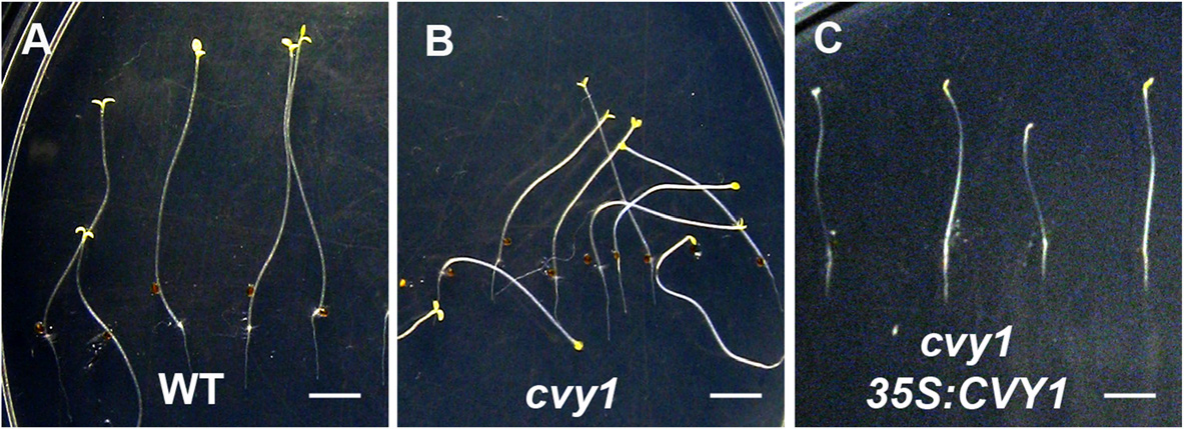
***curvy1*** **mutants displayed distinctly pronounced dark phenotypes. (A, B)** wild type **(A)**, *cvy1* **(B)** and *cvy1 35S:CVY1* rescue **(C)** seedlings grown on agar plates for 12 days after germination in the dark are here depicted. Under dark growth conditions, *curvy1* mutant **(B)** showed lack of vertical growth orientation compared to WT **(A)**. The etiolated dark phenotype was perfectly rescued by overexpressing *CVY1* gene in the mutant background **(C)**. Bars = 5 mm.

We examined the organization of the actin cytoskeleton in pavement cells of *cvy1* mutant. The wild type (n = 8) generates a significantly higher population of polarized actin bundles extending towards to the peripheral patterning of the pavement cells (Figure 5A). The *cvy1* pavement cells (n = 10) displayed the presence of high levels of presumably diffuse and loosely aligned actin monomers and filaments, but lacking in polarized actin bundles (Figure 5B). The actin cytoskeleton phenotype of *cvy1* mutant is similar to what has been reported for *arp2/3* mutants [2]. The actin cytoskeleton phenotype (Figure 5) suggests that the *CURVY1* gene might function in a common WAVE/SCAR2-ARP2/3 pathway [2,3,6,20,30]. To further support the function of *CURVY1* through actin cytoskeleton network, we used the ImageJ analysis tool to quantify the number of actin bundles (AB) in pavement cells after thresholding the stacked image to easily track/count the actin bundles. Using a grid system (of 25 μsq surface area as unit of the grid) covering the entire pavement cell (Additional file 2: Figure S2), we obtained a significantly (P <0.05) higher number of actin bundles in WT (AB = 6.333 ± 1732, n = 9 samples) compared to *cvy1* mutant (AB = 2.333 ± 1414, n = 9 samples) per surface unit of the grid (Additional file 2: Figure S2). Consistent with the diffused and loosely aligned actin cytoskeleton phenotype of *curvy1* (Figure 5), the dark grown *cvy1* mutant displayed a loss of gravity and polarized growth orientation compared to WT (Figure 6A, B). In addition, the etiolated phenotype was rescued by overexpressing *CURVY1* gene in *cvy1* mutant plants (Figure 6C, Table 4), suggesting that *CURVY1* regulates cell morphology and polarized growth through functional actin cytoskeleton network in *Arabidopsis thaliana*.

### *CURVY1* controls other biological processes in plants

**Figure 7:**
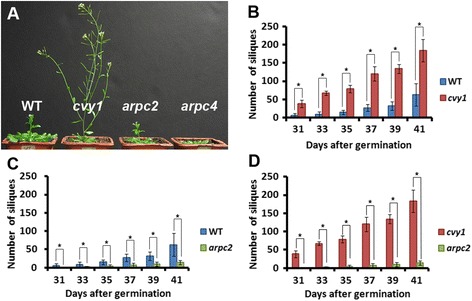
***curvy1*** **mutant flowers earlier and produce more seeds than WT and** ***arp2/3*** **mutants. (A)** Representative growth phenotype of the seedlings is depicted at 29 days after germination in soil. **(B-D)** Number of siliques produced at indicated days after germination. Comparative production of siliques between WT and *cvy1* **(B)**, WT and *arpc2* **(C)**, *cvy1* and *arpc2* **(D)** is depicted. No silique was produced by arpc4 mutant at 41 days after germination and no comparative data was done with *arpc4* mutant. Means ± STDEV of plants (n = 6) per genotype are shown. Significant differences in comparison analysis are indicated with asterisks: *P< 0.05.

Interestingly, we noticed an early flowering phenotype in *cvy1* mutants (n = 18) supported by a significantly (P <0.05) reduced number of rosette leaves (9 ± 0.8) compared to the wild type (13 ± 0.5, n = 16) at bolting time. Unlike *cvy1* mutants, *arp2/3* mutants (*arpc2* rosette leaves = 18 ± 1.3, n = 14 and *arpc4* rosette leaves = 22 ± 1.5, n = 12, at bolting time) showed a significantly (P <0.05) delayed flowering phenotype compared to WT and *cvy1* mutant (Figure 7A), suggesting that CURVY1 might regulate growth development through distinct signal transduction cascades to control transition from vegetative to reproductive stage. The homozygous *cvy1* mutant (n = 9) displayed a faster growth rate and higher seed pod (siliques) production compared to the wild type (n = 9) and *arp2/3* mutants (n = 9) (Figure 7B-D), indicating that CURVY1 negatively regulates cell division and growth in meristemic regions as well as the overall production of seeds. Under similar growth conditions, *cvy1* mutants produced about three-fold more seed pods (siliques: yield) compared to WT (Figure 7B) and 10-fold more siliques than *arpc2* mutant (Figure 7C). Manipulating *CURVY1* gene might be a promising target to improve crop yield in higher plants. To support this observation, we weighed all the seeds of each genotype at harvest time and found *cvy1* seeds weighing two and half times more than those of WT and five times more than seeds of *arpc2* mutant. Our data reveals that mutations in *CVY1* gene result in early flowering, senescence, and improved seed productivity. The mechanism by which CURVY1 regulates transition processes from vegetative to reproductive phase needs to be investigated in agronomically important crops.

## Conclusions

In summary, we present in this work the identification of a new gene, *CURVY1* that regulates growth, cell morphogenesis and seed production in *Arabidopsis thaliana*. This work presents evidence that *CURVY1* belongs to the “distorted group” of genes. Homozygous *cvy1* mutant displayed strong morphological phenotypes that are indistinguishable from the well-characterized *DISTORTED* trichome mutants [2]. The *CURVY1* gene encoding a receptor-like protein kinase is ubiquitously expressed in all tissues tested. The distorted trichome phenotype in *cvy1* mutant was rescued by expressing *CURVY1* gene in the mutant background. Unlike the other *DISTORTED* mutants, mutation of *CURVY1* gene promotes early flowering and seed production in *Arabidopsis thaliana*. Overall, *CURVY1* represents a novel receptor-like kinase gene involved in regulating cell morphogenesis, including trichome and pavement cell shape patterning through local actin cytoskeleton assembling and additionally functions in signal transduction cascades that control flowering time and seed production in plants.

## Methods

### Plant strain, growth conditions and mutant characterization

*Arabidopsis thaliana* (ecotype Col-0) and *cvyt1* knockout mutant (T-DNA SALK_018797) [from Arabidopsis Biological Research Center (ABRC)] were used throughout this work. Appropriate seeds were sown on Murashige and Skoog (1× MS) agar plates or soil and seedlings were allowed to grow under continuous illumination (120–150 μEm^−2^ s^−1^) at 24°C. For *cvy1* mutant characterization, T-DNA insertion was PCR-confirmed using *CVY1* gene specific primers (Table 1) and T-DNA left border primer Lb (Table 1). To analyze the expression of *CVY1* gene in mutant backgrounds, total RNA was extracted from the homozygous T-DNA insertion mutants by TRIzol reagent (Molecular Research Center) and then reversed transcribed using qScript cDNA Supermix (Quanta BioSciences, Gaithersburg, MD, USA) as previously described [30]. Thereafter, the cDNA was used as template for PCR using *CVY1* gene-specific primers (Table 1), running 30 amplification cycles (linear range of amplification) [30]. PCR fragments were separated on 1% agarose gels containing ethidium bromide. A cDNA fragment generated from ACTIN served as an internal control.

For complementation test, a RT-PCR amplification of 2600 bp fragment containing the 5’ and 3’ untranslated regions as well as *CVY1*-encoding sequence (At2g39360) from WT cDNA (Table 1) was cloned into the *Sma*I site of the pROK2 vector [43] in front of CaMV 35S promoter-driven overexpression [43,44] and stably transformed *cvy1* mutant background by the floral dip method [29]. For tissue specific gene expression analysis, the cDNA from respective tissues was used to perform real-time qPCR of CVY1 gene expression. Real-time qPCR was performed on Eco real-time PCR system (Illumina, San Diego, CA, USA) using PerfeCTa SYBR green FastMix (Quanta BioScience, Gaithersburg, MD, USA). The relative *CVY1* expression level was assessed using *ACTIN* gene as internal control (Table 1).

### *Arabidopsis thaliana* CrRLK1-like family: structural characterization and phylogenetic analysis

*Catharanthus roseus* RLK (CrRLK) characteristics were used to retrieve the 17 members of *Arabidopsis thaliana* CrRLK1-like gene family according to Hematy and Hofte [31] and used to generate the phylogenetic tree according to Gachomo et al. [30]. CURVY1 (a member of CrRLK1-like family) protein functional domains were studied using different structure-functional motifs and/or patterns databases such as Pfam v25.0 (pfam.sanger.ac.uk), Prosite (prosite.expasy.org/scanprosite) and Conserved Domain Database (CDD) v3.02, CDART (Conserved Domain Architecture Retrieval Tool) to reveal the kinase catalytic domains, the carbohydrate, substrate and ATP binding sites and their 3D structural features according to Gachomo et al. [30].

### Scanning electron microscopy (SEM)

SEM images of upper developing leaves, showing mature trichomes of WT and *cvy1* mutant were acquired at different magnifications as previously described [30]. SEM images were taken using a LEO 1450 EP SEM [30].

### Cell morphological analysis

Confocal image analysis was performed on one week after germination of plate grown plants. Pavement-cell shape analysis was performed by staining the samples with 10 μM of the lipophilic dye, FM464, for 2 hr in darkness under rocking conditions. The images were acquired using confocal microscopy (inverted Leica SP8 confocal microscope at 488 nm, 25% laser power and emission at 600 nm). The F-actin localization was done according to Kotchoni et al. [2]. Images were collected using an inverted Leica SP8 confocal microscope with water-immersion objective. The images were processed and analyzed using ImageJ software.

### Determination of flowering time

Flowering time was assessed by counting the number of rosette leaves when flower bolts were 1 cm in length or when floral buds were visible at the center of the rosette as previously reported [30,45].

### Statistical analysis

Experiments were performed at least three times. Data were expressed as mean values ± SE. P values were determined by Student’s t test analysis.

## Additional files

Additional file 1: Figure S1. Expression of *CVY1* in various tissues. qRT-PCR analysis of *CVY1* gene (±SE) of three replicate samples per indicated tissues are depicted. A.U. = Relative expression *CURVY1* using internal Actin control in Arbitrary Unit. Additional file 2: Figure S2.
*CVY1* regulates cell morphogenesis through actin cytoskeleton bundles. Actin filament bundles were analyzed with ImageJ after the image was thresholded to obtain filament bundles instead of monomeric actin subunit in the pavement cell. Actin bundles were quantified per grid (each grid measuring 25 μsp) as depicted in WT Col-0 (A) compared to *cvy1* (B) pavement cells. Actin bundles crossing the grid boundary were counted for both adjacent grids.

## References

[1] Geitmann A (2010). Mechanical modeling and structural analysis of the primary plant cell wall. Curr Opi Plant Biol.

[2] Kotchoni SO, Zakharova T, Mallery EL, El-Din El-Assal S, Le J, Szymanski DB (2009). The association of the Arabidopsis actin-related protein (ARP) 2/3 complex with cell membranes is linked to its assembly status, but not to its activation. Plant Physiol.

[3] Zhang C, Kotchoni SO, Samuels L, Szymanski DB (2010). SPIKE1 signals originate from and assemble specialized domains of the endoplasmic reticulum. Curr Biol.

[4] Hulskamp M, Misera S, Jurgens G (1994). Genetic dissection of trichome cell development in Arabidopsis. Cell.

[5] Uhrig JF, Mutondo M, Zimmermann I, Deeks MJ, Machesky LM, Thomas P, Uhrig S, Rambke C, Hussey PJ, Hulskamp M (2007). The role of Arabidopsis SCAR genes in ARP2–ARP3-dependent cell morphogenesis. Development.

[6] Zhang C, Mallery E, Reagan S, Boyko VP, Kotchoni SO, Szymanski DB (2013). The endoplasmic reticulum is a reservoir for WAVE/SCAR regulatory complex signaling in the Arabidopsis leaf. Plant Physiol.

[7] Mathur J (2005). The ARP2/3 complex: giving plant cells a leading edge. Bioessays.

[8] Smith LG, Oppenheimer DG (2005). Spatial control of cell expansion by the plant cytoskeleton. Annu Rev Cell Dev Biol.

[9] Szymanski DB (2005). Breaking the WAVE complex: the point of Arabidopsis trichomes. Curr Opin Plant Biol.

[10] Hussey PJ, Ketelaar T, Deeks MJ (2006). Control of the actin cytoskeleton in plant cell growth. Annu Rev Plant Biol.

[11] Basu D, El-Assal Sel D, Le J, Mallery EL, Szymanski DB (2004). Interchangeable functions of Arabidopsis PIROGI and the human WAVE complex subunit SRA1 during leaf epidermal development. Development.

[12] Brembu T, Winge P, Seem M, Bones AM (2004). NAPP and PIRP encode subunits of a putative wave regulatory protein complex involved in plant cell morphogenesis. Plant Cell.

[13] Deeks MJ, Kaloriti D, Davies B, Malho R, Hussey PJ (2004). Arabidopsis NAP1 is essential for Arp2/3-dependent trichome morphogenesis. Curr Biol.

[14] Frank M, Egile C, Dyachok J, Djakovic S, Nolasco M, Li R, Smith LG (2004). Activation of Arp2/3 complex-dependent actin polymerization by plant proteins distantly related to Scar/WAVE. Proc Natl Acad Sci USA.

[15] Saedler R, Zimmermann I, Mutondo M, Hulskamp M (2004). The Arabidopsis KLUNKER gene controls cell shape changes and encodes the AtSRA1 homolog. Plant Mol Biol.

[16] Zimmermann I, Saedler R, Mutondo M, Hulskamp M (2004). The Arabidopsis GNARLED gene encodes the NAP125 homolog and controls several actin-based cell shape changes. Mol Genet Genomics.

[17] Zhang X, Dyachok J, Krishnakumar S, Smith LG, Oppenheimer DG (2005). IRREGULAR TRICHOME BRANCH1 in Arabidopsis encodes a plant homolog of the actin-related protein2/3 complex activator Scar/WAVE that regulates actin and microtubule organization. Plant Cell.

[18] Le J, Mallery EL, Zhang C, Brankle S, Szymanski DB (2006). Arabidopsis BRICK1/HSPC300 is an essential WAVE-complex subunit that selectively stabilizes the Arp2/3 activator SCAR2. Curr Biol.

[19] Schwab B, Folkers U, Ilgenfritz H, Hulskamp M (2000). Trichome morphogenesis in Arabidopsis. Philos Trans R Soc Lond B Biol Sci.

[20] Zhang C, Mallery EL, Schlueter J, Huang S, Fan Y, Brankle S, Staiger CJ, Szymanski DB (2008). Arabidopsis SCARs function interchangeably to meet actin-related protein 2/3 activation thresholds during morphogenesis. Plant Cell.

[21] Szymanski DB (2009). Plant cells taking shape: new insights into cytoplasmic control. Curr Opin Plant Biol.

[22] Benschop JJ, Mohammed S, O’Flaherty M, Heck AJR, Slijper M, Menke FLH (2007). Quantitative Phosphoproteomics of Early Elicitor Signaling in *Arabidopsis*. Mol Cell Proteomics.

[23] Gomez-Gomez L, Felix G, Boller T (1999). A single locus determines sensitivity to bacterial flagellin in *Arabidopsis thaliana*. Plant J.

[24] Nuhse TS, Peck SC, Hirt H, Boller T (2000). Microbial elicitors induce activation and dual phosphorylation of the *Arabidopsis thaliana* MAPK 6. J Biol Chem.

[25] Asai T, Tena G, Plotnikova J, Willmann MR, Chiu WL, Gomez-Gomez L, Boller T, Ausubel FM, Sheen J (2002). MAP kinase signalling cascade in Arabidopsis innate immunity. Nature.

[26] Kotchoni SO, Gachomo EW (2006). The reactive oxygen species network pathways: an essential prerequisite for perception of pathogen attack and disease resistance in plants. J Biosci.

[27] Schwab B, Mathur J, Saedler R, Schwarz H, Frey B, Scheidegger C, Hulskamp M (2003). Regulation of cell expansion by the DISTORTED genes in *Arabidopsis thaliana*: actin controls the spatial organization of microtubules. Mol Genet Genomics.

[28] Zimmermann P, Hirsch-Hoffmann M, Hennig L, Gruissem W (2004). GENEVESTIGATOR: Arabidopsis microarray database and analysis toolbox. Plant Physiol.

[29] Clough SJ, Bent AF (1998). Floral dip: a simplified method for Agrobacterium-mediated transformation of Arabidopsis thaliana. Plant J.

[30] Gachomo EW, Jimenez-Lopez JC, Jno Baptiste L, Kotchoni SO (2014). GIGANTUS1 (GTS1), a member of Transducin/WD40 protein superfamily, controls seed germination, growth and biomass accumulation through ribosome-biogenesis protein interactions in *Arabidopsis thaliana*. BMC Plant Biol.

[31] Steinwand BJ, Kieber JJ (2010). The role of receptor-like kinases in regulating cell wall function. Plant Physiol.

[32] Becraft PW (2002). Receptor kinase signaling in plant development. Annu Rev Cell Dev Biol.

[33] Huck N, Moore JM, Federer M, Grossniklaus U (2003). The Arabidopsis mutant feronia disrupts the female gametophytic control of pollen tube reception. Development.

[34] Rotman N, Rozier F, Boavida L, Dumas C, Berger F, Faure JE (2003). Female control of male gamete delivery during fertilization in Arabidopsis thaliana. Curr Biol.

[35] Guo H, Li L, Ye H, Yu X, Algreen A, Yin Y (2009). Three related receptorlike kinases are required for optimal cell elongation in Arabidopsis thaliana. Proc Natl Acad Sci USA.

[36] Deslauriers SD, Larsen PB (2010). FERONIA is a key modulator of brassinosteroid and ethylene responsiveness in Arabidopsis hypocotyls. Mol Plant.

[37] Hematy K, Sado PE, Van Tuinen A, Rochange S, Desnos T, Balzergue S, Pelletier S, Renou JP, Hofte H (2007). A receptor-like kinase mediates the response of Arabidopsis cells to the inhibition of cellulose synthesis. Curr Biol.

[38] Boisson-Dernier A, Roy S, Kritsas K, Grobei MA, Jaciubek M, Schroeder JI, Grossniklaus U (2009). Disruption of the pollenexpressed FERONIA homologs ANXUR1 and ANXUR2 triggers pollen tube discharge. Development.

[39] Miyazaki S, Murata T, Sakurai-Ozato N, Kubo M, Demura T, Fukuda H, Hasebe M (2009). ANXUR1 and 2, sister genes to FERONIA/SIRENE, are male factors for coordinated fertilization. Curr Biol.

[40] Hematy K, Hofte H (2008). Novel receptor kinases involved in growth regulation. Curr Opi Plant Biol.

[41] Lindner H, Muller LM, Boisson-Dernier A, Grossniklaus U (2012). CrRLK1L receptor-like kinases: not just another brick in the wall. Curr Opi Plant Biol.

[42] Shiu SH, Bleecker AB (2001). Receptor-like kinases from Arabidopsis form a monophyletic gene family related to animal receptor kinases. Proc Natl Acad Sci USA.

[43] Kotchoni SO, Kuhns C, Ditzer A, Kirch H-H, Bartels D (2006). Over-expression of different aldehyde dehydrogenase genes in *Arabidopsis thaliana* confers tolerance to abiotic stress and protects plants against lipid peroxidation and oxidative stress. Plant Cell Environ.

[44] Baulcombe DC, Saunders GS, Bevan MW, Mayo MA, Harrison BD (1986). Expression of biologically active viral satellite RNA from the nuclear genome of transformed plants. Nature.

[45] Kotchoni SO, Larrimore KE, Mukherjee M, Kempinski CF, Barth C (2009). Alterations in the endogenous ascorbic acid content affect flowering time in Arabidopsis. Plant Physiol.

