# Supplementary figures and images for: The Arabidopsis CURVY1 (CVY1) gene encoding a novel receptor-like protein kinase regulates cell morphogenesis, flowering time and seed production
Source: BMC Plant Biol. 2014 Aug 27;14:221. doi: 10.1186/s12870-014-0221-7 (PMC4244047; doi:10.1186/s12870-014-0221-7)

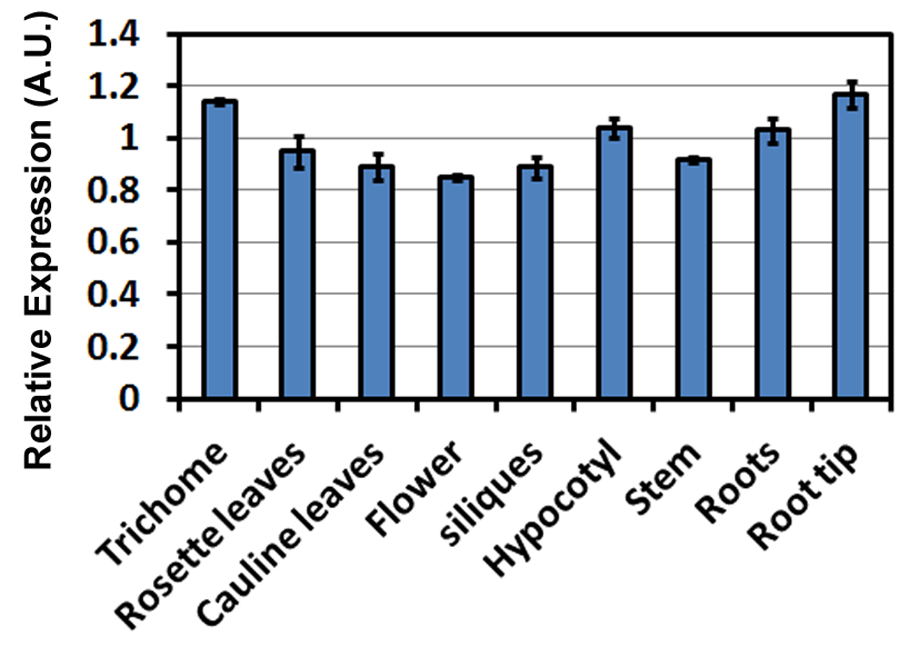

Supplement: Additional file 1: Figure S1. — Expression of CVY1 in various tissues. qRT-PCR analysis of CVY1 gene (±SE) of three replicate samples per indicated tissues are depicted. A.U. = Relative expression CURVY1 using internal Actin control in Arbitrary Unit. [file 12870_2014_221_MOESM1_ESM.tiff]

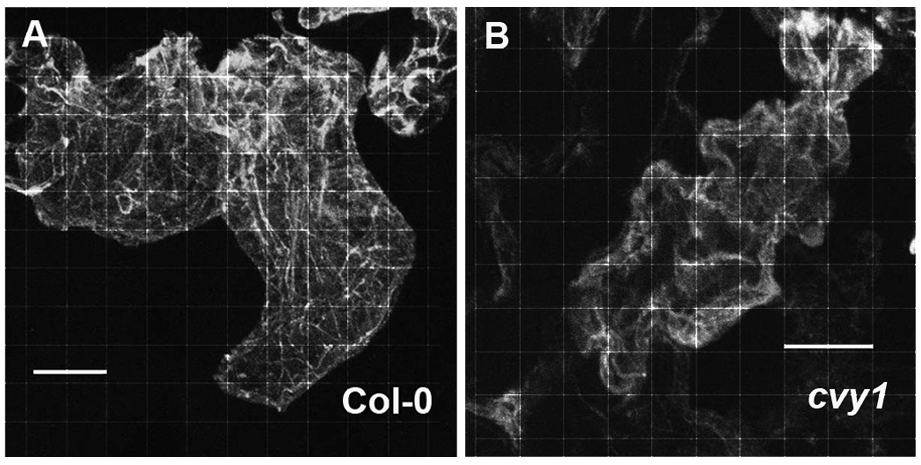

Supplement: Additional file 2: Figure S2. — CVY1 regulates cell morphogenesis through actin cytoskeleton bundles. Actin filament bundles were analyzed with ImageJ after the image was thresholded to obtain filament bundles instead of monomeric actin subunit in the pavement cell. Actin bundles were quantified per grid (each grid measuring 25 μsp) as depicted in WT Col-0 (A) compared to cvy1 (B) pavement cells. Actin bundles crossing the grid boundary were counted for both adjacent grids. [file 12870_2014_221_MOESM2_ESM.tiff]
